# Transcutaneous Electrical Spinal Cord Stimulation to Promote Recovery in Chronic Spinal Cord Injury

**DOI:** 10.3389/fresc.2021.740307

**Published:** 2022-01-04

**Authors:** Candace Tefertiller, Meghan Rozwod, Eric VandeGriend, Patricia Bartelt, Mitch Sevigny, Andrew C. Smith

**Affiliations:** ^1^Craig Hospital, Englewood, CO, United States; ^2^Department of Physical Medicine and Rehabilitation, University of Colorado, Denver, CO, United States

**Keywords:** neuromodulation, spinal cord injury, transcutaneous electrical spinal cord stimulation, task-specific, tetraplegia

## Abstract

**Objective:** To evaluate the impact of using transcutaneous electrical spinal cord stimulation (TSCSTSCS) on upper and lower extremity function in individuals with chronic spinal cord injury (SCI).

**Design:** Prospective case series.

**Setting:** SCI specific rehabilitation hospital.

**Participants:** A convenience sample (*N* = 7) of individuals with tetraplegia who had previously been discharged from outpatient therapy due to a plateau in progress.

**Interventions:** Individuals participated in 60 min of upper extremity (UE) functional task-specific practice (FTP) in combination with TSCS and 60 min of locomotor training in combination with TSCS 5x/week.

**Main Outcome Measures:** The primary outcome for this analysis was the Capabilities of Upper Extremity Test (CUE-T). Secondary outcomes include UE motor score (UEMS), LE motor score (LEMS), sensation (light touch and pin prick), Nine-Hole Peg Test, 10 meter walk test, 6 min walk test, and 5 min stand test.

**Results:** Seven individuals (four motor complete; three motor incomplete) completed 20–80 sessions UE and LE training augmented with TSCS and without any serious adverse events. Improvements were reported on the CUE-T in all seven individuals. Two individuals improved their ASIA impairment scale (AIS) classification (B to C; C to D) and two individuals improved their neurologic level of injury by one level (C4–C5; C5–C6). Sensation improved in five individuals and all four who started out with motor complete SCIs were able to voluntarily activate their LEs on command in the presence of stimulation.

**Conclusion:** Individuals with chronic SCI who had previously demonstrated a plateau in function after an intensive outpatient therapy program were able to improve in a variety of UE and LE outcomes in response to TSCS without any adverse events. This was a small pilot study and future fully powered studies with comparative interventions need to be completed to assess efficacy.

## Background

Evidence in spinal cord injury (SCI) reports the presence of viable fibers traveling across the lesion site in the majority (84%) (1) of individuals who have been clinically diagnosed with a complete SCI (1–3). These individuals are considered to have a “discomplete” profile with subfunctional neural connections that remain intact across the injury site but are not robust enough to generate clinically observable or functional movement. Even though not strong enough to result in voluntary movement, these connections may be able to modulate the excitability of spinal sensorimotor networks below the level of the lesion. Spared supraspinal connections paired with neuromodulatory interventions such as spinal cord stimulation demonstrate potential to upregulate the nervous system to promote motor recovery, even in individuals diagnosed with motor complete SCIs or incomplete SCIs with non-functional motor strength (3, 4). The evidence to support non-invasive transcutaneous electrical spinal cord stimulation (TSCS) as a neuromodulator activating sublesional spinal networks has been growing in recent years (5–10).

TSCS is designed to modulate spinal networks through surface electrodes placed directly over the spinal cord using a unique waveform to deliver high-current electrical stimulation to the posterior aspect of the spinal cord (5). Improvements in upper extremity (UE) grip strength (11, 12) as well as UE function (11) have been reported in response to cervical TSCS, even in individuals with chronic (>1 year) and severe (no motor preservation below the level of the injury) SCIs. Enhanced trunk control and stability (9), improved lower extremity (LE) function, locomotor output, and standing (6, 8, 13) as well as reduced LE spasm severity (14) have been reported in response to training with TSCS focused over the thoracolumbar region. Autonomic function has also been evaluated in multiple studies using TSCS in individuals with SCI with reported improvements in bladder (13, 15) and sexual function along with thermoregulation and cardiovascular function (5). While there is growing evidence (primarily pilot studies) to support that functional training in combination with TSCS may be beneficial for individuals with SCI, many knowledge gaps remain including, but not limited to, who may benefit most from this intervention.

Even though published results with TSCS in individuals with severe SCIs have been promising, this impact has not yet been evaluated in those who have already completed an intensive outpatient (OP) therapy program focused on recovery-based interventions. The primary objective of this pilot study was to evaluate the safety and feasibility of using TSCS in a clinical setting to promote recovery after SCI in a cohort of individuals recently discharged from OP therapy due to a plateau in recovery.

## Methods

### Participants

Participants were recruited for this study from a convenience sample of individuals who had previously completed at least 40 sessions of OP therapy (total of 3 h per day of UE and LE training) focused on recovery-based interventions LE training focused on improving standing and stepping—using a treadmill with 30% body weight support (BWS) followed by 30 min of over ground stepping training. UE training included 1.5 h/day of functional electrical stimulation (FES) in combination with functional task specific practice (FTP) to the UEs and trunk using 12 channels of stimulation 5 days/week. Full UE protocol described in Tefertiller et al.

Other inclusion criteria consisted of the following: 18 years or older; history of traumatic cervical SCI, completed outpatient rehabilitation program and discharged due to a plateau in progress; absence of complicating physical or mental conditions as determined by their physician that would preclude the individual from safely using electrical stimulation; intact skin; and using a wheelchair as a primary means of mobility (>50% of day). Individuals were excluded if they Individuals were excluded from participating in the study if they had a recent history of fracture, contractures, pressure injuries, deep vein thrombosis or urinary tract or other infections that might interfere with interventions; Unstable or symptomatic, chronic cardiac or respiratory complaints; received Botox injections within the last 3 months; using anti-spasm medications; received stem cell therapy within 2 years prior to enrollment; and pregnant.

Participants were chosen to participate in the current study because they received intensive OP therapy (described above) and were discharged from therapy due to a plateau in recovery of function in either or both upper and lower extremity outcomes. Outcome assessments focused on UE and LE recovery were completed before beginning the study and at every 20 sessions of participation. All assessments were evaluated without stimulation. Participants were discharged from the study at a 20-session interval if unable to demonstrate progress on at least one outcome measure, but remained in the study if continuing to demonstrate improvements, up to 80 sessions. Participants provided written informed consent for study participation, which was approved by the local Institutional Review Board.

### Transcutaneous Electrical Spinal Cord Stimulation (TSCS) Protocol

**Figure 1:**
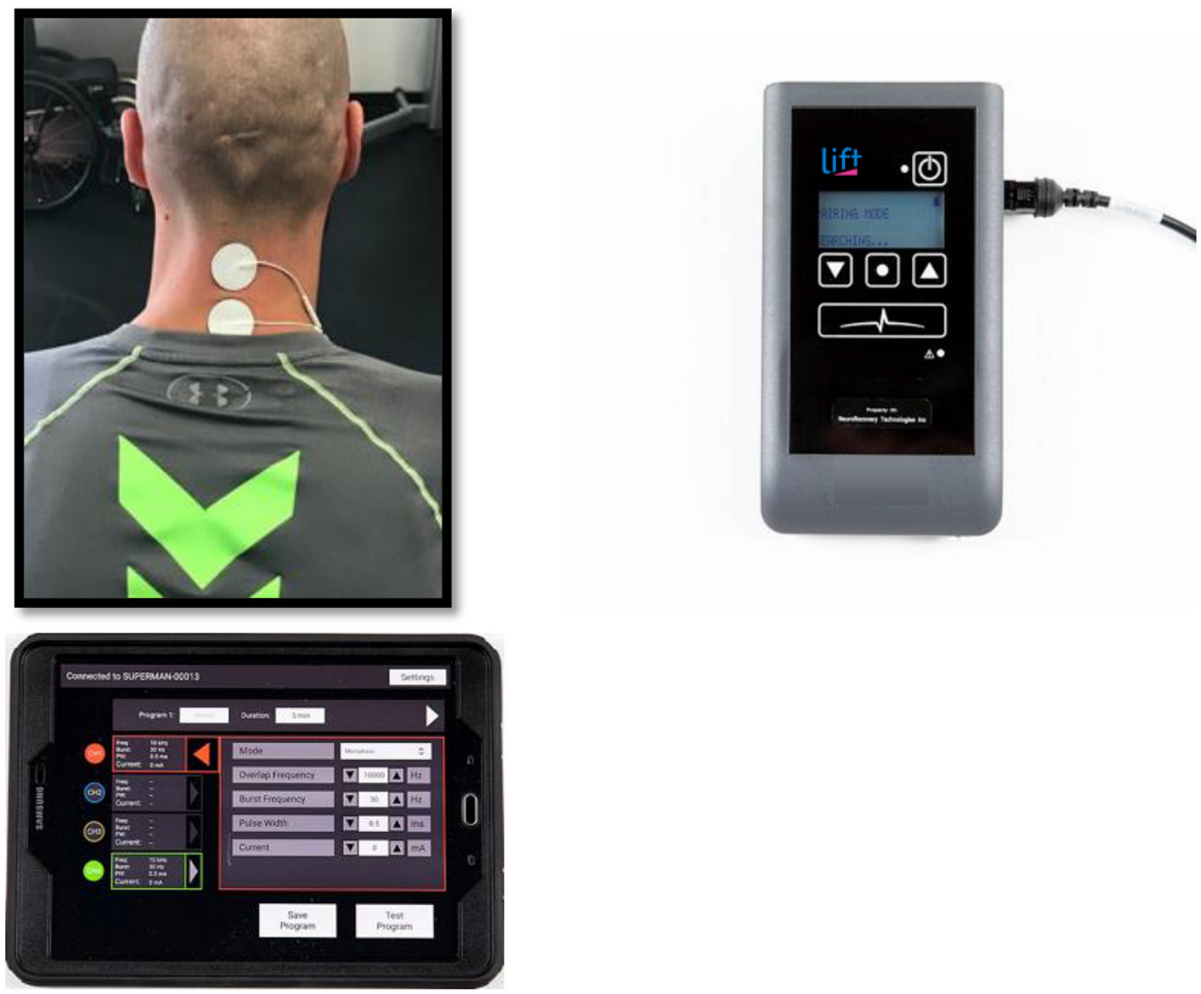
Transcutaneous electrical spinal cord stimulation set up at the cervical spine for one representative participant.

This study was carried out using a non-invasive TSCS stimulator (NeuroRecovery Technologies Inc.). During each UE study session, self-adhesive 1.25″ round electrodes (Axelgard, Inc.) were placed midline on the skin at two sites between spinous processes C3-C4 and C6-C7 as cathodes, see Figure 1 (11). Two anodes (3 × 5″ oval electrodes) were also placed symmetrically over the iliac crests. TSCS was delivered using monophasic rectangular 1 ms pulses at a frequency 30 Hz with each pulse filled by a carrier frequency of 10 kHz. Stimulation was delivered during LE training in the exact same manner except the stimulation sites were midline between spinous processes T11-T12 and L1-L2 (13). Stimulation amplitude was slowly increased by 1 ma increments to reach the voluntary activation threshold (VAT) which was defined as the amplitude at which optimal functional movement of the targeted intervention area (UE or LE) was achieved in a joint below the level of injury. Amplitude was re-assessed each time a participant changed to a new functional activity during each session and the amplitude was adjusted to a level that provided optimal functional movement for that specific activity.

### Intervention

All participants completed 1.5 h of UE and 1.5 h of LE training/day ×5 days/week for at least 20 sessions. UE training consisted of 60 min of FTP in combination with TSCS over the cervical spinal cord (C3-C4, C6-C7) followed by 15 min of functional training without TSCS. UE training was focused on the following activities: Overhead press; can open and fine motor manipulation; door pull; open with key; grasp and release; and shoulder flexion. LE training consisted of standing and stepping training—over ground (OG) or using a treadmill with body weight support (BWS). Three therapists provided assistance for stepping on the manual treadmill and over ground training. Participants were encouraged to step independently and assistance was only provided at the trunk and LEs when they were unable to do so safely.

For individuals unable to initiate stepping independently, training included 60 min of over group step initiation and stand training in combination with TSCS. For individuals able to independently initiate a step with either LE, training included 60 min of treadmill training with 30% body weight support (BWS) in combination with TSCS followed immediately by 15 min of over ground stand and walking training without TSCS.

### Outcome Measures

The International Standards for Neurological Classification of SCI (ISNCSCI) assesses motor, sensory, and neurologic impairment after SCI. Excellent intrarater reliability has been reported in chronic SCI for pin prick, light touch and motor scores (16). Total upper extremity motor score (UEMS) is the total of left and right side muscle strength graded from 5 UE muscles on a 0 (total paralysis) to 5 (normal active movement) scale while the same grading is used for 5 LE muscles to achieve the total lower extremity motor score (LEMS) on the ISNCSCI exam (17). The ISNCSCI is reliable and valid (18, 19) in SCI. The minimally clinically important difference (MCID) published for the ISNCSCI total motor scores is 4.48; total sensory sore is 5.19; UEMS is 2.72; and LEMS is 3.66 (20).

The Capabilities of Upper Extremity—Test (CUE-T), an assessment that measures the amount of difficulty experienced in performing specific actions with one or both arms and hands. The CUE-T has 32 items, each scored on a 0–4 point scale with total scores ranging from 0 to 128 (higher scores demonstrating greater UE function) along with right/left subscale scores (21). The CUE-T has been shown to valid, reliable (22), and responsive in SCI (21). The published MCID for the CUE-T Total score is 12.0 (21). Nine-Hole Peg Test measures finger dexterity after neurologic injury. Scores are based on the time required to move 9 pegs to a wooden board and captured in seconds. This assessment has shown excellent test-retest reliability in chronic stroke (23), but psychometric properties have not been reported in SCI.

The 10 meter walk test (10MWT) assesses the amount of time required to walk 10 m with or without an AD and velocity is reported in meters per second. The 10MWT has excellent test-retest reliability in SCI (24). The MCID for the 10MWT is 0.06 m/s (25). The 6 min walk test (6minWT) assesses distance walked over 6 min as a submaximal test of aerobic capacity and endurance with the score reported in meters. Excellent inter-rater and intra-rater reliability has been demonstrated using the 6minWT in SCI (26, 27). The minimal detectable change (MDC) for the 6minWT is 45.8 m (28). The purpose of the Overground Standing Test is to determine how long an individual can stand unassisted for up to 5 min while bearing weight through their lower extremities and minimizing UE weight bearing. This test has not been validated in SCI, but is common practice in clinical care.

### Data Analysis

All sample summary data are represented as raw scores for each individual. Since this sample consists of only seven individuals, statistical significance was not considered and all analyses are exploratory. Demographic and injury information was reported from baseline and includes age, height, weight, level of education, time since injury (TSI) in months, injury etiology, level of injury (LOI), and AIS (American Spinal Injury Association [ASIA] Impairment Score). The number of completed sessions was reported at NRN and Transcutaneous for both UE and LE. All outcome data are displayed at both baseline and final evaluation. The change in each outcome measure was calculated from baseline to the individual's final evaluation.

## Results

Seven individuals with chronic (>1 year) SCI American Spinal Cord Injury Association (ASIA) ASIA impairment scale (AIS) A-D completed this study with the number of study sessions ranging from 20 to 80 among the group. The majority were 30 years of age or younger (Mean = 27.7, SD = 13.5), ranging from 18 to 55 years. See Table 1 for demographic, injury, and session information. There were no serious adverse events or unexpected adverse events in this study. All adverse events (AEs) were minor in nature (skin issues) that resolved within 24–48 h and did not prevent training participation.

**Table 1:** Individual's demographic, injury, and session information at baseline.

	**Age**	**TSI (Months)**	**Sex**	**LOI**	**AIS**	**OP**		**Time between OP and TESS (months)**	**TESS**				**Lesion Length (mm)**
				**Baseline/** **final evaluation**	**Baseline/** **final evaluation**	**UE sessions**	**LE sessions**		**UE Sessions**	**UE Amplitude (ma)**	**LE Sessions**	**LE Amplitude (ma)**	
1	20	24	Male	C6/C6	B/B	60	40	15	20	70–150	20	160–190	35.3
2	30	15	Female	C5/C4	B/C	80	80	8	60	65–100	60	145–190	24.9
3	18	16	Female	C4/C4	C/C	60	60	6	60	45–50	60	80–95	13.6
4	20	26	Male	C6/C6	B/B	80	80	15	20	75–150	20	175–190	48.6
5	33	38	Male	C4/C5	B/B	80	40	33	20	50–75	20	140–170	52.4
6	55	19	Male	C5/C6	D/D	80	80	10	60	65–75	60	75–100	32.7
7	18	18	Male	C4/C4	C/D	80	80	9	80	45–65	80	30–90	16.9

*TSI, Time since Injury; LOI, Level of Injury; AIS, ASIA (American Spinal Injury Association) Impairment Score; OP, Outpatient Therapy; TESS, Transcutaneous Electrical Spinal Stimulation; UE, Upper Extremity; LE, Lower Extremity, mm: Millimeter*.

Table 2 displays the change results for each outcome measure collected. Within each outcome, the majority of the sample showed improvements (Table 3). The UE and LE motor change scores ranged from an 11-point increase to a 3-point decrease. Sensation (combined light touch and pin prick) improved in five participants with one individual who demonstrated a 43 point change. Every participant that had complete data improved their CUE-T, nine-hole peg test, 10MWT, and 6minWT. For the Overground Stand Test, three participants had a positive change while the remaining four showed no change.

**Table 2:** Baseline to post-TESS outcome change.

	**Time point**	**UEMS**	**LEMS**	**Light touch** **+** **pin prick**	**CUE-T total score**	Nine-hole peg test* **(Peg/s)**	**10 meter walk test** **(m/s)**	**6-min walk test** **(m)**	**5-min stand test**
1	Baseline	23	0	91	45	–	–	–	0:00
	Final evaluation	22	0	105	47	–	–	–	0:00
	Change	−1	0	+14**	+2	–	–	–	0:00
2	Baseline	21	0	96	48	–	–	–	0:00
	Final evaluation	26	1	109	52	0.017	–	–	1:45
	Change	+5**	+1	+13**	+4	-	–	–	+1:45
3	Baseline	10	9	110	19	0.017	–	–	0:26
	Final evaluation	17	11	119	25	0.064	–	–	2:16
	Change	+7**	+2	+9**	+6	+0.047	–	–	+1:50
4	Baseline	24	0	48	53	–	–	–	0:00
	Final evaluation	25	0	46	63	–	–	–	0:00
	Change	+1	0	−2	+10	–	–	–	0:00
5	Baseline	20	0	57	40	0.105	–	–	0:00
	Final evaluation	20	0	56	42	0.167	–	–	0:00
	Change	0	0	−1	+2	+0.062	–	–	0:00
6	Baseline	43	38	123	90	0.153	0.15	50.60	2:45
	Final evaluation	40	40	166	91	0.155	0.31	74.80	5:00
	Change	−3	+2	+43**	+1	+0.003	+0.17	+24.20	+2:15
7	Baseline	27	25	109	63	0.084	0.16	78.24	5:00
	Final evaluation	38	27	120	77	0.143	0.26	88.08	5:00
	Change	+11**	+2	+11**	+14**	+0.059	+0.10	+9.84	0:00

UEMS, Upper Extremity Motor Score; LEMS, Lower Extremity Motor Score; CUE-T, Capabilities of Upper Extremity Test; *Dominant Hand; s: Second; m, Meter; **Exceeded Minimally Clinically Important Difference for this measure.

**Table 3:**
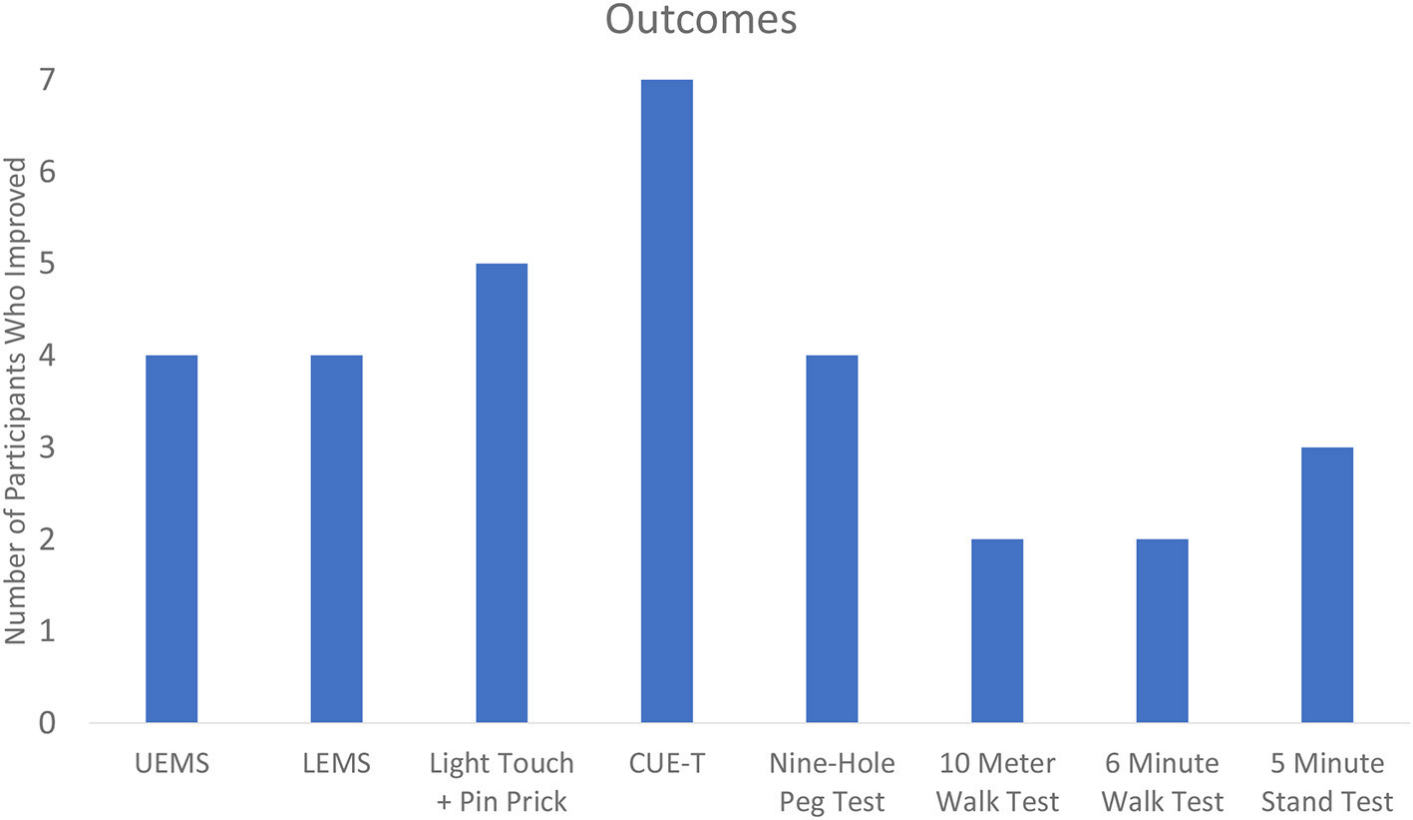
Improvement in outcomes among the sample.

## Discussion

Following a functional recovery plateau with intensive outpatient therapy, seven individuals with chronic spinal cord injury participated in functional task-specific practice training augmented with TSCS in a clinical setting without experiencing any serious adverse events. TSCS demonstrated good clinical utility and was successfully implemented in the clinical setting. Importantly, all seven participants enrolled in this pilot study had previously received intensive OP UE and LE training and had demonstrated a plateau in recovery prior to enrollment, yet positive changes were observed in UE strength, UE function, LE strength, and sensation in response to training augmented with TSCS.

In terms of UE recovery, the two participants who started the study with an AIS C classification demonstrated the most significant changes in UEMS exhibiting 7 and 11 point improvements which far exceeds the MCID for this measure of 2.72 (20). Only one participant with an AIS B classification demonstrated an UEMS improvement that exceeded the MCID for this measure (5 point change) while all others demonstrated no improvement or a slight decline. This suggests that those with more incomplete injuries (AIS C) may demonstrate a better response to this intervention than those with more complete injuries, but results should be interpreted with caution due to the limited sample size. In regards to UE function, (CUE-T), only one participant (07) demonstrated a changed that exceeded the MCID for this measure of 12.0 points (21). None of the participants met the MCID for LEMS [3.66 points (20)]; however, the two individuals with AIS C injuries, exceeded the MCID for the 10MWT (0.10 m/s) (28). Of note, all four individuals with AIS B injuries were able to demonstrate isolated voluntary activation of LE muscle groups in the presence of stimulation. One individual who began the study as an AIS B, transitioned to a C and was able to stand with a walker without physical assistance at study completion.

Although exact mechanisms behind TSCS are not fully understood, upregulation of somatosensation is thought to play a critical role, with much of the effect attributed to direct modulation of the dorsal roots (3). Changes in sensory function exceeding the MCID (5.19) (20) for this measure were found in 5 out of the 7 individuals who completed the trial with change scores ranging from 9 to 43. This suggests that spared sensory pathways in both the dorsal (light touch) and anteriolateral (pin prick/crude touch) aspects of the spinal cord were engaged and at least some sensory recovery was facilitated in the majority of participants who completed this trial. Gad et al. (12) also reported an average improvement in sensation of 8.4 ± 2.9 points in 6 individuals who received cervical TSCS focused on improving UE strength and function.

Inanici et al. (11) reported substantial improvements in the Graded Redefined Assessment of Strength, Sensation and Prehension (52 points), UEMS (10 points), and pinch strength in a 62 year old male diagnosed with a C3 SCI (AIS D) who received TSCS in combination with intensive physical therapy over 9 weeks (mean duration was 60 ± 20 min/session of stimulation). In agreement with these findings, one of our participants demonstrated improvements that exceeded the MCIDs for UEMS, the CUE-T and total sensation who was also diagnosed with an AIS D injury, which may help explain the higher ceiling for recovery experienced by these two individuals. However, the type of training individuals participated in prior to TSCS was substantially different which may also impact the outcomes. The case study by Inanici et al. (11) reported participating in regular exercise-based therapy 4–5 times/week prior to TSCS, but was not reported to include surface electrical stimulation. All seven individuals included in this study had received an average of 72 UE retraining sessions augmented with 12 channels of FES in OP therapy prior to receiving TSCS. In addition, four out of seven individuals in this case series exhibited changes that exceeded the MCID for the CUE-T between their initial and final evaluations in OP therapy in response to task specific training augmented with FES. Therefore, some of the participants in this case series may have reached their ceiling of UE recovery using FES prior to receiving TSCS, while others continued to demonstrate improvements from the addition of TSCS.

TSCS has also been shown to improve LE function and standing ability after SCI (5, 13). Gerassimenko et al. (5) reported on five individuals with motor complete SCI who regained locomotor-like stepping movement in their lower extremities in response to TSCS combined with lower extremity training. In this study, all four individuals diagnosed with motor complete SCIs (AIS B) were able to activate their LEs in the presence of TSCS, upon voluntary command. See Supplementary Video 1 of a participant diagnosed with a C6 AIS B who demonstrated a strong trunk extension response when stimulating for LE training during his initial session of TSCS therapy with an amplitude of 100 ma indicating this therapy also positively impacted trunk activation during LE training. Supplementary Video 2 demonstrates the ability this participant (1) diagnosed with a C6 AIS B SCI to activate his left LE on command also during his initial session of TSCS. Stimulation was increased to 100 ma and the participant (1) was asked to extend his left leg which he was able to do on command. In Supplementary Video 3 after 20 sessions of training, this same participant (1) was asked to extend bilateral LEs as quickly as possible and then asked to stop the movement on command. This increase in activation may suggest improved neuronal network strength in response to 20 training with TSCS. Participant 2 transitioned from an AIS B to a C during the study and was able to stand with a walker and without physical assistance after completing 60 sessions of training. Supplementary Video 4 demonstrates the ability for participant 2 to lift her left hip against gravity at 18 sessions of training without stimulation demonstrating her transition from AIS B to C. Supplementary Video 5 depicts participant 4 (C6 AIS B) extending both LEs on command with a stimulation amplitude of 110 ma. Supplementary Video 6 depicts participant 5 (C5 AIS B) extending his left leg on command with an amplitude of 120 ma. Both participants who were ambulatory when starting the study were able to improve their speed beyond the MCID for this measure, but the same magnitude in change for walking endurance was not seen.

Completion of this protocol required a significant commitment from study participants (3 h/day at a frequency of 5 days/week). Anecdotally, participants reported they really enjoyed the training and did not want it to end. We did not have anyone drop out of the study and we had very few missed study sessions. Although time intensive, these individuals had previously participated in an OP therapy program that was just as time intensive (3 h/day) so they were well-accustomed to this type of schedule and highly motivated to participate in recovery-based therapies.

## Future Directions

**Figure 2:**
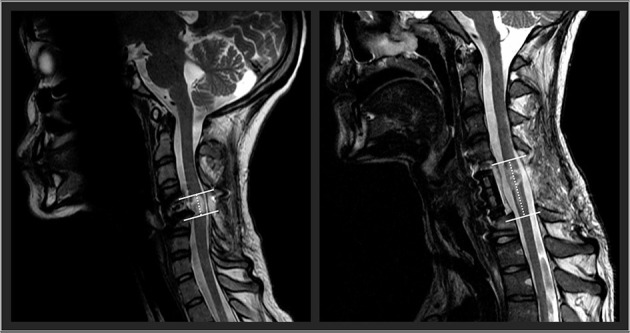
Two representative participants' midsagittal T2 weighted MRIs are shown. The cranial-caudal lesion boundaries are identified in white lines, while the lesion lengths are depicted in the dotted lines. On the left panel, this participant had a relatively shorter lesion length compared to the participant's lesion in the right panel.

Future research should examine the influence of spinal cord lesion characteristics on the responsiveness to TSCS. In an exploratory approach, using our participants' available clinical sagittal T2-weighted magnetic resonance imaging, we found that spinal cord lesion length was negatively correlated with participants' outcomes. High correlations were found with UE motor change scores (*r*_s_ = −0.64), LE motor change scores (*r*_s_ = −0.85), and the nine-hole peg test (*r*_s_ = −0.77). With further research and refinement, lesion length (Figure 2) could be used to identify optimal responders and stratify participant groups for prospective investigation of TSCS. The hope is that with an increased participant sample size using high resolution MRI in a prospective investigation, future research could investigate how lesion length and other measures of spinal cord damage such as widths of midsagittal tissue bridges (29–34), lesion volumes (35), atlas-based estimates of tract damage (36–38) relate to responsiveness to spinal cord stimulation. This work is underway.

## Limitations

This study was a case series with a small sample size. The number of study sessions was not standardized which resulted in a variable dose of training. The sample was heterogeneous in regards to AIS classification and injury level making it difficult to draw any substantial conclusions and limit generalizability of these findings. Although all participants diagnosed with a motor complete injury were able to voluntarily activate lower extremity movement in the presence of stimulation, no specific outcome measures were used to quantify this change.

## Conclusion

Augmenting UE and LE training using TSCS in the clinical setting was shown to be safe and feasible in seven individuals with SCI who had already received intensive physical therapy and plateaued with UE and LE recovery. UE and LE benefits were demonstrated in this sample even after they had already participated in intensive OP therapy, demonstrating that there may be a greater ceiling of recovery for some individuals beyond currently available clinical interventions. Lesion length may be a useful MRI measure to identify optimal TSCS responders. Future research should focus on predicting who will benefit most from TSCS while also identifying those who may need more invasive approaches to maximize recovery.

## Data Availability Statement

The original contributions presented in the study are included in the article/Supplementary Material, further inquiries can be directed to the corresponding author/s.

## Ethics Statement

The studies involving human participants were reviewed and approved by Advarra. The patients/participants provided their written informed consent to participate in this study. Written informed consent was obtained from the individual(s) for the publication of any potentially identifiable images or data included in this article.

## Author Contributions

CT: principal investigator of this study and was the primary author of this manuscript. MR: study coordinator and assisted with manuscript writing. EV: provided the intervention and assisted with manuscript writing. PB: primary data collector and assisted with manuscript writing. MS: statistician and assisted with manuscript writing. ACS: provided the MRI data/interpretation and assisted with manuscript writing. All authors contributed to the article and approved the submitted version.

## Funding

ACS was supported by an NIH Award, K12 HD055931 Comprehensive Opportunities in Rehabilitation Research Training.

## Conflict of Interest

The authors declare that the research was conducted in the absence of any commercial or financial relationships that could be construed as a potential conflict of interest.

## Publisher's Note

All claims expressed in this article are solely those of the authors and do not necessarily represent those of their affiliated organizations, or those of the publisher, the editors and the reviewers. Any product that may be evaluated in this article, or claim that may be made by its manufacturer, is not guaranteed or endorsed by the publisher.
